# Bilateral neck fracture in bimodular femoral stem after primary total hip arthroplasty: a case report

**DOI:** 10.1186/s12891-021-04210-y

**Published:** 2021-04-16

**Authors:** Samo K. Fokter, Nenad Gubeljak, Jožef Predan, Jure Sevšek, Jan Zajc, Zmago Krajnc

**Affiliations:** 1 Department of Orthopaedic Surgery, University Medical Centre, 5 Ljubljanska street, SLO-2000 Maribor, Slovenia; 2 Faculty of Mechanical Engineering, University of Maribor, 17 Smetanova street, SLO-2000 Maribor, Slovenia; 3 Slovenj Gradec General Hospital, 1 Gosposvetska road, SLO-2380 Slovenj Gradec, Slovenia

**Keywords:** Bi-modular femoral stem, Case report, Modular femoral neck, Neck fracture, Taper corrosion, Titanium alloy, Total hip arthroplasty complication

## Abstract

**Background:**

Bi-modular stems were introduced in primary total hip arthroplasty (THA) to enable better control of the femoral offset, leg length, and hip stability. Despite numerous reports on modular femoral neck fractures, some designs are still marketed worldwide. While the risk factors for the sudden failure are multifactorial and mostly known, the timing of this new THA complication is not predictable by any means.

**Case presentation:**

In this report, the literature regarding one of the most popular bi-modular stems with specific neck-stem coupling (oval Morse taper) is reviewed and illustrated with a case of bilateral modular neck fracture in a patient with idiopathic aseptic necrosis of femoral heads treated with primary bi-modular THA. Because of bilateral modular femoral neck fracture, which occurred 3 years on the left side and 20 years after implantation on the right side, the patient required a total of 6 revisions and 208 days of hospitalized care.

**Conclusion:**

To our knowledge, this is the first report of bilateral modular neck fracture in a single patient. Even though the same surgeon performed both operations and used the same neck length and orientation, fractures occurred with a 17-year time difference after implantation. This shows that we cannot predict with certainty when a fracture might occur. Orthopaedic surgeons should use bi-modular stem designs for primary THA very cautiously.

## Background

The bi-modular femoral stem is a recent innovation in primary total hip arthroplasty (THA) that enables the orthopaedic surgeon a more anatomically correct restoration. Single-centre studies reported excellent results with titanium alloy made stems and modular necks even in the long term [1]. However, some producers were forced to remove their implant from the market or change the neck material to cobalt-based alloys due to an increase of titanium alloy modular femoral neck failures [2].

Different types of corrosion can develop on the stem-neck junction. Studies have linked implant failures to mechanically assisted crevice corrosion (MACC), which also includes the most common damage process fretting corrosion [3]. Corrosion products can cause adverse local tissue reactions that lead to THA failure [4].

Several orthopaedic studies on bi-modular THA’s have identified risk factors that can lead to modular neck fracture. These include time since implantation, a long neck, a cobalt-chromium alloy neck, younger age, male sex, and higher BMI [4].

This is the first report of a case with bilateral titanium-titanium alloy modular neck fracture in a patient treated with primary THA using current-generation stem designs with 9 × 18 mm rectangular cross-section Morse taper as a method of intraoperative stem-neck fixation (Microport Orthopedics Inc., Arlington, TN, USA, formerly Cremascoli Ortho, Milan, Italy). Proximally, the Ti6Al4V modular neck was equipped with standard 12/14 mm cylindrical taper for head-neck coupling. Distally, the rectangular vertices were rounded by fillets; taper angles in the flat and curved sections were machined to 4°. This type of distal taper geometry was not changed even though femoral stem’s design changed with different manufacturers. With its three different neck versions (straight, valgus/varus and ante/retro; both deviations for 8° or 15°) and two different neck lengths (short and long), it enables the surgeon to properly restore anatomical conditions in most patients [5].

## Case presentation

A 38-year-old male with body mass index (BMI) 31.5 kg/m2 was presented in 1998 with more than 12 months of progressive pain in both hips. Radiographs and computer tomography scanning revealed avascular necrosis of the femoral heads (Ficat stage IV on the right and Ficat stage III on the left side). The right hip was treated with an uncemented THA through a standard posterior approach. A screw-in titanium-alloy (TiAl6V4) acetabular cup size 56 mm with polyethylene insert (RCM, Cremascoli Ortho) was inserted and the used femoral component was a size 6 proximally plasma-spray hydroxyapatite-coated, and anatomically shaped titanium-alloy modular stem (An.C.A. Fit, Cremascoli Ortho) with long straight titanium modular neck of the same material and 28-mm, + 3.5 mm (L) length cobalt-chromium (Co-Cr) femoral head. Next year, the patient’s left hip was treated through a standard posterior approach by the same orthopaedic surgeon with a 60 mm screw-in titanium-alloy acetabular cup with polyethylene insert (RCM, Cremascoli Ortho) and the size 7 femoral component (An.C.A. Fit, Cremascoli Ortho) with long straight titanium modular neck and 28-mm alumina ceramic (Biolox Forte, CeramTec, Plochingen, Germany) femoral head with + 3.5 mm (L) length. The same surgeon performed both surgeries and the patient recovered from both with no postoperative complications.

**Fig. 1:**
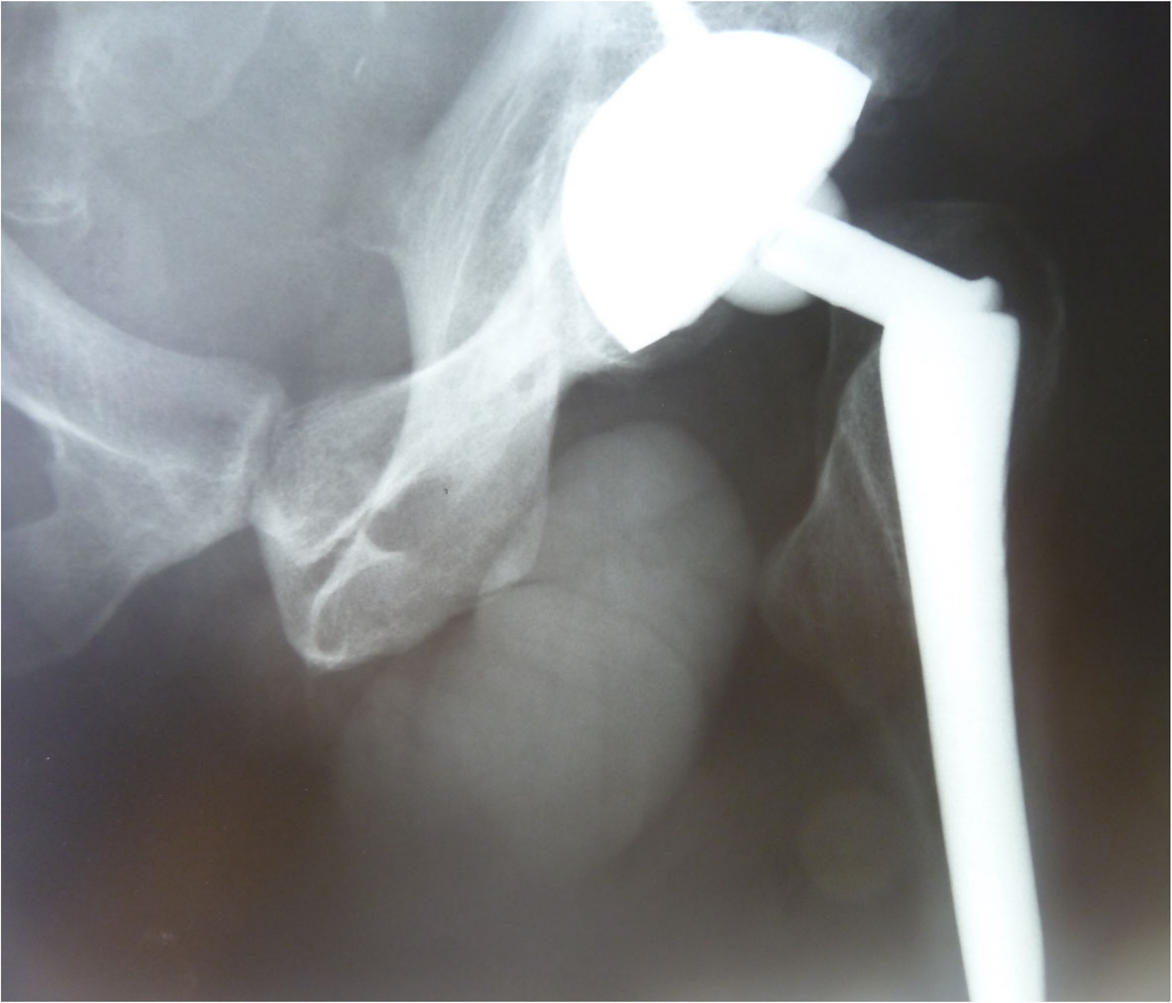
Emergency room X-ray of the left hip with fractured modular femoral neck 3 years after primary THA

Three years after primary left THA, the patient presented with sudden left groin pain and inability to bear weight on the affected leg. Radiographs revealed a fracture of the left modular femoral neck (Fig. 1). At revision, no signs of polyethylene wear, local tissue adverse reaction or pseudotumor formation were present. However, it was impossible to disengage the modular neck from the femoral taper. An extended trochanteric osteotomy was performed to remove the original implant and a Wagner-type revision modular stem (Limacorporate S.p.A., Udine, Italy) was inserted. Tissue samples were sent for bacteriology and have remained sterile. However, the patient underwent subsequent revision surgeries for stem subsidence, instability and eventually for *Staphyloccocus Epidermidis* infection. The infection was eliminated through a two-staged procedure and the patient was finally successfully reconstructed in 2017 with Alloclassic Zweymüller SL stem, Continuum acetabular component and Biolox Delta ceramic femoral head (Zimmer Biomet). Radiographs taken 2 years after revision demonstrated stable fixation of all THA components of the left hip.

**Fig. 2:**
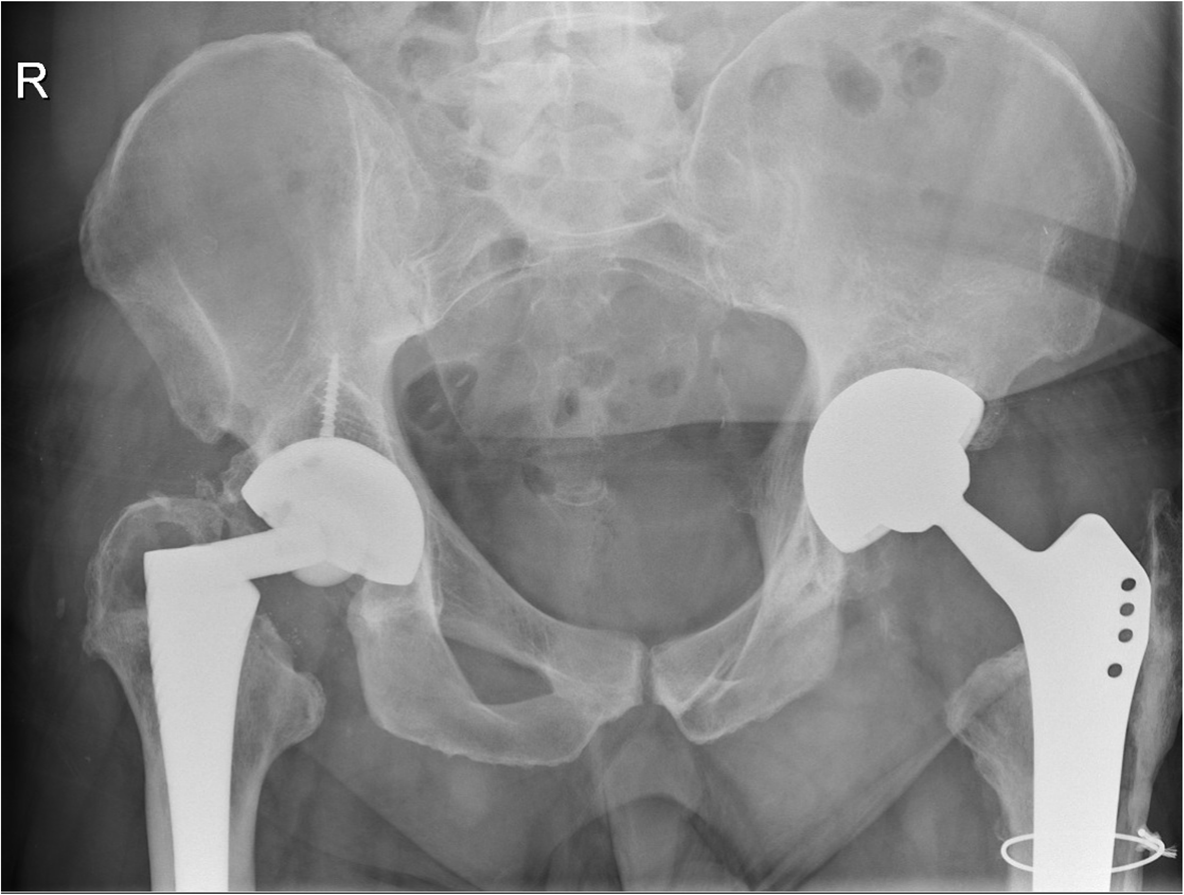
Pelvic X-ray with modular neck fracture shown on the right and situation 1 year after fourth revision of the left THA, respectively

**Fig. 3:**
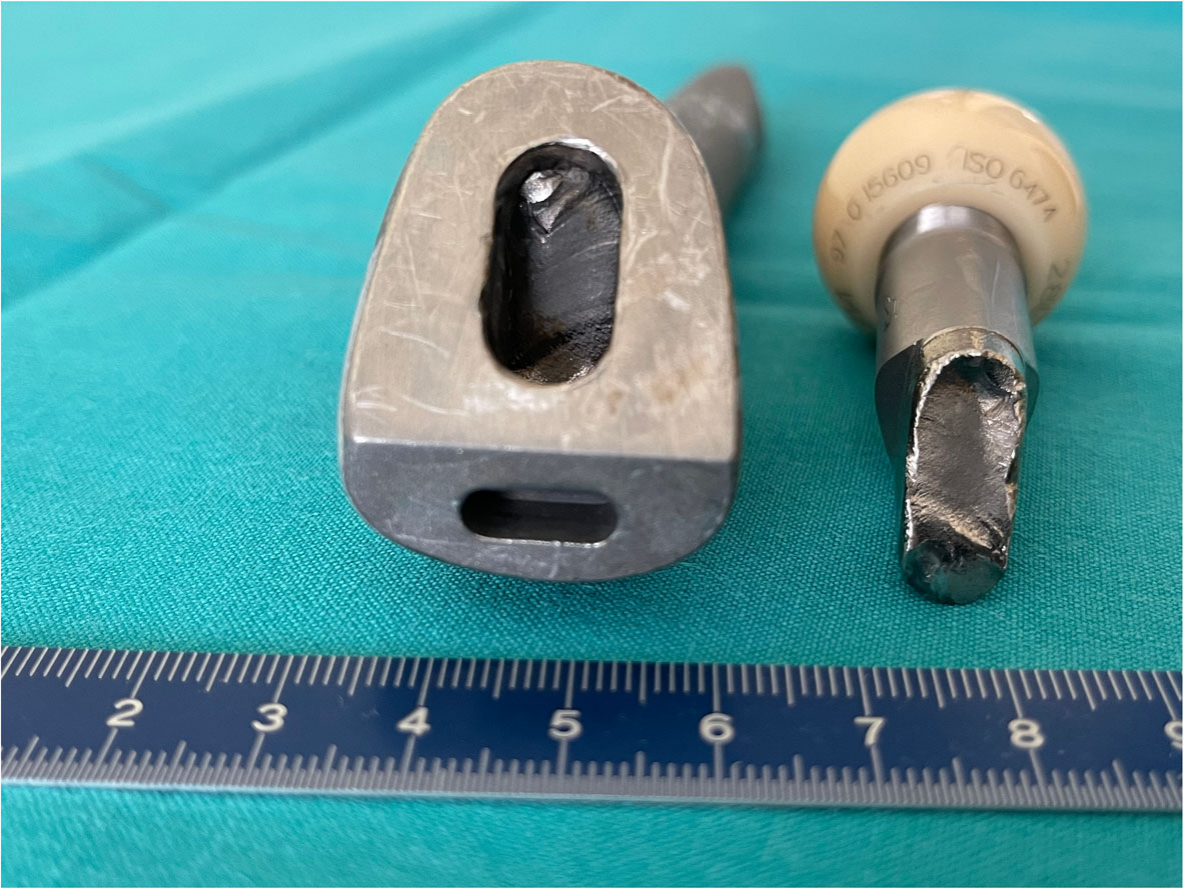
Optical photograph of the fractured surface of the modular neck with the distal part still engaged in the conical cavity of the femoral stem

Unfortunately, 20 years after the right THA insertion, the patient presented again to the Emergency Clinics with a fracture of the right-side modular neck (Fig. 2). At revision, no significant local tissue adverse reaction or pseudotumor formation were present. Again, it was impossible to disengage the modular neck from the femoral taper **(**Fig. 3**)**. The femoral component was extracted via a single longitudinal proximal splitting and two-chisel technique. The acetabular component was revised as well due to excessive polyethylene wear. A replacement liner was not available. The hip was reconstructed with a long Wagner-type revision stem (Limacorporate), Continuum acetabular component (Zimmer Biomet) and ceramic femoral head (Biolox Delta). Tissue samples taken during revision surgery and sent for cultures remain sterile. Immediate postoperative course was uneventful and patient was discharged as usual. However, he was readmitted 1 month after surgery with signs of deep wound infection. The revision procedure was debridement, antibiotics and implant retention (DAIR) with exchange of femoral head and liner only. Operative cultures found *Cutibacterium (Propionibacterium) acnes*. The patient was treated with antibiotics for 3 months.

**Fig. 4:**
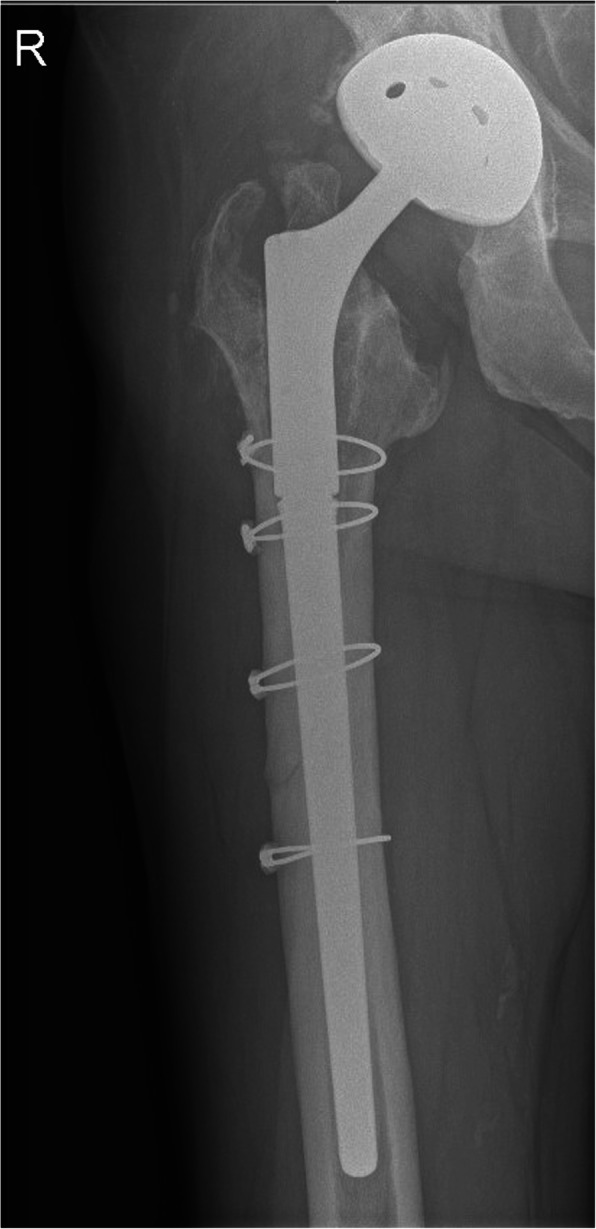
X-ray of the right hip 14 months after last revision

14 months after the last revision, the patient was complaining of mild occasional pain and ambulated without walking aids or shoe lift. The patient’s Harris Hip Score (HHS) was 84 for the right hip and 87 for the left hip, respectively. His University of California at Los Angeles (UCLA) activity score (min 0 - max 10) was 4. The SF-36 outcome scores for general health, health change, pain, physical functioning, and limitations due to physical health were 75, 50, 60, 45 and 25%, respectively, 40% each for social functioning, emotional wellbeing and energy/fatigue, and 0% for role limitations due to emotional problems. The radiographs demonstrated stable fixation of all THA components of both hips (Fig. 4).

## Discussion

Bi-modular stem designs offer surgeons an intraoperative choice of neck version and length independently of the stem size, which has led to their worldwide popularity. There are important differences in femoral neck length, shaft diameter, caput-column-diaphysis (CCD) angle, neck version, and neck offset, and modularity enables the orthopaedic surgeon to adapt to those differences [1]. Excellent results with titanium-titanium alloy made stems and modular necks were reported in single-centre studies [5]. Despite these advantages, several studies reported an increase in titanium alloy modular femoral neck failures [2, 6, 7]. In a study of two groups of THA patients, Duwelius et al. looked for differences in anatomical hip restoration and revision rates between nonmodular and modular stems [8]. A total of 284 patients with nonmodular stems (Zimmer M/L Taper) and 594 patients with modular stems (Zimmer M/L Taper Kinectiv) were followed up with a mean of 2.4 years (maximum 5.9 years). Clinical and radiographic measurements of leg length and offset were done before and after surgery. Clinical evaluations included Harris Hip and SF-12 scores, respectively. With no differences in outcome scores, the authors concluded that modular stems offered no added value over standard non-modular stems [8].

Different specific types of corrosion at the stem-neck junction were found responsible for exchangeable neck failures. In 2014, Mumme et al. submerged standardized alloys (TiAl6V4, CoCr29Mo, FeCrNiMoMnNbN and pure titanium) in human serum and measured in vitro serum levels of metal ions [9]. Elevation of in vitro ions concentration shows that electrochemical corrosion occurs without the need for mechanical load [9]. Types of corrosion that can develop in orthopaedic implants include uniform corrosion attack, galvanic corrosion, fretting corrosion, crevice corrosion, pitting corrosion, intergranular corrosion, leaching, and stress-corrosion cracking [10]. MACC at stem-neck junction was shown to release metal debris that caused local tissue reactions like those found in failed THA-s [11–14].

Titanium alloy is suitable for non-modular prosthesis due to its passive oxide layer that protects it from uniform corrosion. Fretting corrosion is caused by oscillating micro motion between two surfaces [15]. In modular stem prosthesis, micro movements at the stem-neck junction occur and the oxide layer falls off, exposing titanium alloy to bodily fluids [16] Preventing micromotion is thus the best way to avoid damage within modular junctions. Since Co-Cr alloy has a twice as high module of elasticity compared to titanium alloy, there should be less micro movement than with titanium alloy exchangeable necks. However, with neck fracturing already at 2 years after implantation because of an added galvanic corrosion, Co-Cr necks have not proven to be a safe alternative [7].

Other factors that may escalate fretting corrosion include patient BMI, lateral offset, varus femoral stem positioning, longer necks and larger heads, time since implantation and inconsistency in the assembly of modular heads including the force of impaction, the vector of the applied force, and contamination of the interface [3]. A crack can start on the medial proximal side of the neck-stem taper surface [17]. After the initial crack is made, the corrosion can continue without additional external loads on the femoral neck. This type of autocatalytic corrosion is a result of chemical changes within the crevice fluid [17].

Several studies report complications after implantation of bi-modular femoral stems with symmetrically oval Morse taper joints in primary THA. Grupp et al. followed 5000 bi-modular Metha Short Hip stems (Aesculap AG, Tuttlingen, Germany) implanted between 2004 and 2006 and found that 1.4% of the titanium alloy necks failed after 2 years [2]. Bernstein et al. reported 86% clinical failure rate of the Rejuvenate bi-modular stem implant with Co-Cr neck (Stryker Orthopaedics, Mahwah, New Jersey) at 4.2 ± 0.6 years mean final follow up [18]. Pour et al. followed 277 patients after Profemur (successor of An.C.A. Fit prosthesis with the same oval Morse taper neck-stem junction) bi-modular series stem implantation and found 6% neck fractures at 50 months mean follow up [19]. Finally, Kovač et al. studied the long-term behaviour of the Profemur Z modular stem on a national basis (2767 hips followed) and found out that the mean time for bi-modular femoral neck fracture (0.83%) was 4.7 years (SD ± 2.2 years) [4]. Furthermore, long neck, Co-Cr modular neck, which was introduced by the producer (Wright Medical Technology, now MicroPort Orthopedics Inc.) in 2010 to reduce the failure rate of titanium alloy made necks, and male gender represent the independent risk factors for modular neck fracture [4]. In contrast, Pelayo-de-Tomás et al. found only 1 modular neck fracture in their cohort of 317 consecutive patients followed for 6.1 (range, 2–8) years after bi-modular stem (H-MAX M, Lima, San Danielle, Italy) THA [20]. The authors attributed the discrepancy between the H-MAX M model and other commercially available modular stems to the elliptical dual radius neck-stem coupling causing fever micro-movements [20].

In 2009, Blakey et al. conducted a study on 319 patients that were treated with primary THA using uncemented hydroxyapatite-coated An.C.A. Fit modular femoral stem due to osteoarthritis [21]. Five years after implantation, the authors clinically and radiographically examined 212 males and 107 females with a mean age of 64.4 years. Oxford Hip Score got significantly better (from mean 41 points to 20 points). There were two cases of aseptic loosening. They concluded that there were no clinical or radiographic complications due to modularity [21]. In 2017, Toni et al. reported on clinical and radiographic outcomes of 235 patients 13–18 years after An.C.A. Fit prosthesis implantation with ceramic-on-ceramic (CoC) bearing and noted no modular neck fracture; 93.2% of the implanted THA were still functioning well [22]. However, this case report demonstrates that titanium-titanium alloy modular-neck implants in primary THA are not so uniformly good and complication-free.

Lex et al. recently published a systemic review of 14 studies (12 case series and 2 joint registry analyses) on current-generation primary THA using titanium-titanium alloy modular-neck implants, including An.C.A. Fit [23]. The mean follow-up duration of the studies was 5.7 years and they included 591,025 patients, of which 21,841 underwent modular neck THAs and 569,184 received a fixed-neck prosthesis. Even though the authors have found the overall mean revision rate (3.95%) and the mean revision rate for fracture of the modular neck component (0.43%) for modular prosthesis acceptable, they consider modular prostheses to be a viable management option only in patients with considerable anatomical hip deformities that cannot be corrected with standard fixed-neck implants. The review also points out two national registry reports, which revealed that modular neck prostheses had a higher revision rate compared to traditional, fixed-neck prostheses in patients with osteoarthritis [24].

To our knowledge, the present is the first reported case with bilateral bi-modular femoral neck fracture in a single patient. The modular neck fracture in the presented case occurred relatively early on the left and later on the right side. Beside the variation in the size of acetabular and stem components, the only difference between the implants was in the bearing couple, which was ceramic-on-polyethylene (CoP) on the left side and metal-on-polyethylene (MoP) on the right side. Together with slight variation between implant position and offset, these dissimilarities could be responsible for different timing at which modular neck fracture occurred. However, Frisch et al. have shown that the shape of the neck and stem tapers deviate from ideal design dimensions, contributing to relative motions between the neck and the stem and may place the implants at higher risk for failure [24]. This means that such catastrophic failures can occur unpredictively in otherwise normally functioning implant(s).

The patient in this report was a younger active male with a BMI in the obese range. He had a long modular neck implanted in combination with a long head – all of which are known risk factors for modular neck fracture and therefore make the patient at risk for neck failure [5, 7, 10, 19]. The fatigue strength of TiAl6V4 is about 400 MPa. In heavier and more active patients this threshold can be exceeded because very high tensile stresses occur on long necks in combination with long femoral heads [10, 25, 26]. This stress can open microcracks on the surface and stress fields inside the prosthesis add to crack propagation after its initiation. The patient was hospitalized for 208 days altogether due to bilateral bi-modular prosthesis failure, which profoundly impacted his social life and exposed him to other health problems and threats that arise with more prolonged hospitalizations. Since higher septic complications after THA revision surgeries are well established, modular neck fracture per se could not be blamed for septic complications that developed after revisions of both sides in presented patient [27, 28]. However, more complications also mean higher costs of treatment, which is not beneficial for the health care system. If a high percentage of bi-modular stems started to fail, this could become a major public health issue. Continued usage of bi-modular stems for primary THA is therefore not only a problem associated with more treatment complications and patient suffering but also an economic problem and a threatening public health concern.

In conclusion, the patient could have avoided all the unnecessary complications and had a better quality of life if he were primarily treated with monoblock stems instead.

## Data Availability

This is a case report of a single patient; to protect privacy and respect confidentiality, none of the raw data has been made available in any public repository. The original operation reports, intraoperative photographs, imaging studies and outpatient clinical records are retained as per normal procedure among the medical records of our institution. All data concerning the case are presented in the manuscript.

## Abbreviations

THA: Total hip arthroplasty
MACC: Mechanically assisted crevice corrosion
BMI: Body mass index
TiAl6V4: Titanium-aluminium-vanadium
Co-Cr: Cobalt-chromium
DAIR: Debridement, antibiotics and implant retention
HHS: Harris Hip Score
UCLA: University of California at Los Angeles
SF 36: Short form 36
CCD: Centrum-collum-diaphyseal
MPa: Megapascal
CoC: Ceramic-on-ceramic
MoP: Metal on polyethylene
CoP: Ceramic on polyethylene
